# Characteristics of surface water quality and stable isotopes in Bamen Bay watershed, Hainan Province, China

**DOI:** 10.1371/journal.pone.0245438

**Published:** 2021-01-22

**Authors:** Julan Guo, Yilei Yu, Gaojie Wu, Muyuan Ma

**Affiliations:** 1 Institute of Wetland Research, Chinese Academy of Forestry, Beijing, China; 2 Beijing Key Laboratory of Wetland Services and Restoration, Beijing, China; Soil and Water Resources Institute ELGO-DIMITRA, GREECE

## Abstract

Bamen Bay is located at the intersection of the Wenjiao River and Wenchang River in Hainan Province (China), where mangroves have been facing a threat of water quality deterioration. Therefore, it is imperative to study the characteristics of the surface water quality on a watershed scale. Water samples were collected three times from 36 monitoring sites from 2015 to 2016. It was found that nitrate was the main inorganic nitrogen form and all the surface water types were alkaline. Meanwhile, aquaculture water had high content of nitrogen, total phosphorus, chlorophyll a (*Chl*.*a*), total organic carbon (TOC), and chemical oxygen demand (COD). Significant spatial and temporal variations were found for most parameters. However, stable isotopes of δD and δ^18^O indicated that river water mainly originated from atmospheric precipitation and experienced strong evaporation. The water chemistry and isotopes of the Bamen Bay, mangroves, and aquaculture water were initially affected by the mixing of fresh water and seawater, followed by evaporation. The river and reservoir water chemistry were mainly controlled by water–rock interactions and cation exchange as deduced from the ionic relationships and Gibbs plots. These interactions involved the dissolution of calcite-, bicarbonate-, carbonate-, and calcium-containing minerals. Oxidized environments (river, reservoir, and Bamen Bay) were conducive for nitrification, while anaerobic conditions (mangrove and aquaculture water) were beneficial to the reduced nitrogen forms.

## Introduction

Rivers carry dissolved elements and suspended solids from various sources and/or tributaries, and deposit them in different locations or transfer them to lakes or oceans [1–3]. Therefore, rivers play a major role in the global water cycle. River water chemistry or quality also provides information on chemical weathering processes on a basin scale, and reveals the dissolved elements cycle in the continent-river-ocean system [4–7]. Given the complexity of river systems, the geochemical processes are controlled by many factors. Surface water chemistry in river basins is usually impacted by natural factors and anthropogenic activities. The natural factors, include rainfall, temperature, runoff discharge, and weathering processes [3, 8, 9]. Meanwhile, the hydrochemistry of rivers is controlled by climate, lithology, topography, and vegetation [4, 10, 11]. Temporal variation and fluctuations in the river discharge have enormous effects on its hydrochemistry especially in semi-arid and arid rivers [12, 13]. Lithological weathering is generally caused by a complex set of interactions between the lithosphere, atmosphere, hydrosphere, and biosphere [14, 15]. However, evaporites, carbonates, and silicates are the normal weathering sources [7, 14, 16]. In addition, rivers are currently facing severe threats from various anthropogenic activities [3, 17, 18], which include point sources (domestic and industrial effluents) and diffuse sources (urban and farmlands) [6, 19]. Nevertheless, rivers are highly heterogeneous, especially in their physiochemical compositions at spatial and temporal scales [20, 21]. Spatio-temporal patterns of river water chemistry contributes to the understanding of the hydrologic functioning of the river systems [22, 23], which is typically controlled by complex interactions [6].

Water chemistry is mainly determined by various physical, chemical, and biological processes in river systems [4, 7, 24, 25]. The most abundant ions in the water originate from terrestrial and atmospheric systems. These are mainly calcium, magnesium, sodium, potassium, bicarbonate, sulfate, chloride, and nitrate [26]. As a result, elemental ratios and stoichiometry of solutes in the water can indicate the above-mentioned mechanisms controlling the hydrochemistry, and provide qualitative information on the sources through analyzing the different combinations of dissolved cations and anions in the water [4, 7, 27, 28]. In addition, the influence of anthropogenic activities on river water can also be investigated through the ionic relationships [29, 30]. For example, the Gibbs diagram, a plot of TDS versus the weight ratio of Na^+^/(Na^+^ + Ca^2+^) or Cl^−^/(Cl^−^ + HCO_3_^−^), can provide information on the relative importance of the three important mechanisms controlling natural water chemistry in three distinct fields, i.e., rock dominance, evaporation dominance, and precipitation dominance areas [4].

Water chemistry of the world’s largest rivers has received more attention due to their global significance [4, 31, 32]. Therefore, the natural controls of river chemistry at a global scale have been widely studied [27]. These studies have been done for large river systems in the tropics, such as Amazon [33], Congo [34], and Orinoco [35]. However, studies performed on smaller watersheds in the tropics are rare, especially for rivers with mangroves flowing into ocean. Bamen Bay basin, located in Wenchang city, Hainan Province, is composed of two rivers: the Wenjiao and Wenchang River. Bamen Bay mangrove is centered on the four shoals of the bay, and radiates several kilometers upstream of the two rivers. In recent decades, shrimp ponds, with an area of about 4 km^2^, were constructed in the large-scale shoals, which accounts for approximately 13% of the mangrove [36, 37]. Mangrove areas are important to the ecosystem, however, their number is declining around the world [38]. Their health is also currently facing a significant decline [39], due to a large input load of ammonium, phosphorus, and heavy metals from upstream cities and aquaculture [40, 41]. The most prevalent and serious problems for coastal ecosystems are poor estuary water quality and eutrophication [42–44]. In the past few decades, the amount of human nutrients transported by input channels such as, rivers to mangroves has increased significantly [44]. Aquaculture development is one of the greatest threats to the mangrove ecosystems [45, 46], as it is established by clearing the mangroves [47]. The water quality in adjacent mangrove ecosystems is also significantly affected by shrimp wastewater [48, 49], that is rich in particulates and dissolved organic and inorganic nutrients [50–53]. The cumulative effects of these pollutants are directly proportional to the emissions and nutrient concentrations [54, 55]. According to our knowledge, river water chemistry in the Bamen Bay basin has not been studied before. Therefore, the study of a typical tropical watershed helps to improve on the understanding of river water chemistry evolution.

The main objectives of this study are as follows: (1) clarifying the water chemistry and hydrogen and oxygen stable isotope composition of the surface water in the Bamen Bay watershed, (2) ascertaining the temporal and spatial variations of the surface water chemistry and stable isotopes, and (3) determining the main controlling factors of surface water chemical composition.

## Materials and methods

### Study site

**Fig 1:**
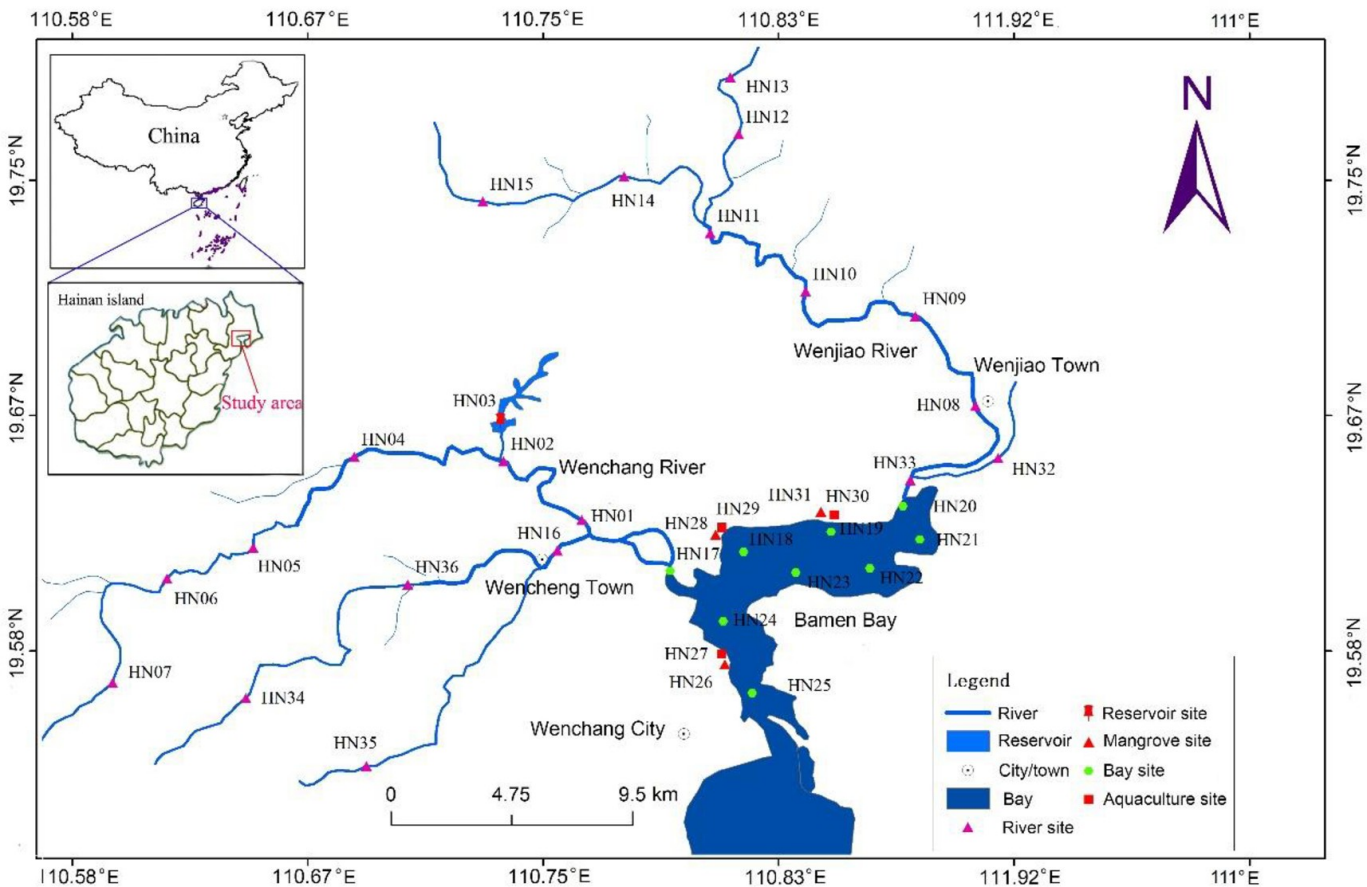
Location of study site and sampling stations in Bamen Bay watershed. Map of Bamen Bay watershed are illustrated by authors using ArcGis Software. Map and shapefile of China and Hainan Province are from Map Standard Service system (http://bzdt.ch.mnr.gov.cn/), and there is no copyright issue.

The study site is in Wenchang City (north east of Hainan Island), Hainan Province, China. This city has a total population of 560,000 people (2016) (Fig 1). Topographically, the study area is a low hilly plain with an average altitude of 42.55 m, with the terrain sloping from the southwestern inland to the northeastern coast. The flat terrain in the north east is part of the plain terrace with an altitude of below 50 m, while the southwestern terrain, with an altitude of 50 to 150 m, was undulating and part of the low hilly platform [56–58].

The climate is typical of a tropical monsoon island, since it is part of the coastal zone of the northern tropical margin that has excellent light, water, humidity, and heat resources. Frost and distinct seasons (spring, summer, autumn, and winter) are not found throughout the year. The annual temperature, sunshine, and humidity averages are 23.9°C, 1953.8 hours, and 87%, respectively. Annual rainfall is abundant, at 1721.6 mm (1529.8–1948.6 mm), dividing the climate into dry and wet periods. The rainy season runs from May to October, accounting for 80% of the yearly rainfall [59].

Wenjiao River and Wenchang River are the two main rivers on the Bamen Bay watershed. Wenjiao, with a total length of 56 km and a drainage area of 529.85 km^2^, is in the eastern part of Wenchang County. It originates from Shudetou, Qiongshan County, and flows to the south east through Wenjiao Town. Meanwhile, Wenchang, that has many tributaries, originates from Dou Niuling in the Qiongshan District, Haikou City, and its mainstream flows south east through the old town of Wenchang City. It has a total length of 37 km and drainage area of 381 km^2^. The average depth of the river upstream and downstream is about 1.0 and 2.5 m, respectively. The depth of Bamen Bay ranges from 3 to 8 m. Qinglan Port Mangrove Wetland Nature Reserve located along the coast of Bamen Bay, with a total area of 20,199 km^2^ (mangrove area: 8,354 km^2^; tidal flat and water area: 11,845 km^2^), is the second-largest in China. The muddy and deep coastal soil is suitable for mangrove growth. The tide is an irregular full-day tide, with the highest (2.38m) and lowest (0.01 m) tide level in Bamen Bay [36, 60]. The wastewater from the fish and shrimp culture, with area of 7.6 km^2^, is discharged directly into the mangroves.

### Methods

#### Water sampling

The Bamen Bay surface water, includes river, reservoir, mangrove, aquaculture, and bay water. Mangrove water is the water that is stored inside the mangrove, which is a mixture of fresh water and sea water. Aquaculture water refers to water from shrimp farming. To investigate the water chemistry and isotope compositions, samples along the river and in other water types were collected three times during November 2015, March and August 2016. Monitoring stations of river, mangrove, aquaculture, and Bamen Bay were given in Fig 1.

#### Analytical techniques

The pH, water temperature (T, °C), electrical conductivity (EC), dissolved oxygen (DO), and oxidation-reduction potential (ORP/Eh, mV) were measured in situ using a multiparameter portable meter (HACH40d, USA) supplied by Hach Company (Loveland, CO, USA). All water samples were collected at 0.5 m below the water surface using two polyethylene bottles and preserved at 4°C. They were then taken back to the laboratory and analyzed within 24 hours.

Water samples for chemical and isotopic analysis were filtrated through a 0.45 μm filter (Millipore cellulose acetate membrane) before any laboratory analysis. Bicarbonate (HCO_3_^−^) was determined by titration using sulfuric acid (0.02 mol/L) until the endpoint as dictated by methyl orange indicator was reached. Potassium permanganate index (COD_Mn_), total organic carbon (TOC), and chlorophyll a were measured according to the Water and Wastewater Monitoring and Analysis Method [61].

The main anions, including fluoride (F^−^), chloride (Cl^−^), sulfate (SO_4_^2−^) and nitrate (NO_3_^−^), were measured using an ionic chromatograph (Thermo Fisher ICS2100) made by DIONEX (Sunnyvale, CA, USA), with a detection limit of 0.01 mg/L. The main cations, including potassium (K^+^), sodium (Na^+^), calcium (Ca^2+^), magnesium (Mg^2+^), and other trace metals, were measured by inductively coupled plasma spectroscopy (ICP-OES) (5300 DV, PerkinElmer), with a detection limit of 0.01 and 0.01 μg/L (trace elements). Ammonia nitrogen (NH_3_-N), nitrite nitrogen (NO_2_-N), and soluble total phosphorus (STP: 0.001 mg/L) were measured using a Smartchem 200 batch analyzer in AMS, with a detection limit of 0.01 mg/L, made by Alliance (Paris, France). Stable oxygen and hydrogen isotopes in the water were determined using a liquid-water isotope analyzer (DLT-100, Los Gatos Research Inc., USA), where the ratio accuracy of O^18^/O^16^ and H^2^/H^1^ isotope was 0.2‰ and 0.6‰, respectively.

#### Data analysis

A balance error of less than 5% was required on the cation and anion data before it was used for further analysis. Otherwise, the measurements were redone until the equilibrium error was achieved. The graphs of the data were plotted using Origin 8.5 software from Origin Lab Corporation (Hampton, MA, USA).

The negative logarithm of electron activity is represented by pE calculated in Eq (1) as follows [62, 63]:

$$pE=\frac{EhF}{2.303RT}$$ (1)

where, F is Faraday constant (96.42 kJV^−1^ g^−1^ equivalent), R is the gas constant (8.314 Jmol^−1^ deg^−1^), and T is the absolute temperature in Kelvin.

## Results and discussion

### General water quality and stable isotopes

General water chemistry and stable isotopes of the surface water are reported in S1 Table. pH ranged from 7.94 to 9.43, showing that the all the water systems were alkaline, with the order of pH as: reservoir > aquaculture > mangrove > Bamen Bay > river. The water temperature (T) ranged from 27.0 to 29.7°C, with the highest value found in the reservoir. Meanwhile, the EC ranged from 337 to 39257 μS/cm in the order of Bamen Bay > mangrove > aquaculture > river > reservoir. The river and reservoir water were both fresh water, while the others were seawater. Positive ORP values showed oxidizing power in the order of river > Bamen Bay > reservoir > aquaculture > mangrove. DO ranged from 5.66 to 10.88 mg/L, in the order of reservoir > aquaculture > Bamen Bay > river > mangrove. The downstream river was more influenced by agricultural activities and domestic wastewater compared to the upstream reservoir. The same order of cations in the Bamen Bay, mangrove, and aquaculture was found: Na > Mg > K > Ca, while the orders in the river and reservoir were Na > Ca > Mg > K, and Na > K >Ca > Mg, respectively. The order of anions in the river and reservoir was similar: Cl > HCO_3_^-^ > SO_4_^2-^, while that in others was Cl > SO_4_^2-^ > HCO_3_^-^. The order of Cl and SO_4_^2-^ in different water systems was also the same, in the order Bamen Bay > mangrove > aquaculture > river > reservoir. Meanwhile, all the water had high Na and Cl content.

The nitrogen form order (NO_3_–N > NH_3_–N > NO_2_–N) was consistent in the five water types, with NO_3_–N recognized as the main inorganic nitrogen. The orders of these nitrogen form were NO_3_–N: Bamen Bay > mangrove > aquaculture > river > reservoir, NH_3_-N: mangrove > aquaculture > Bamen Bay > river > reservoir, and NO_2_-N: aquaculture > Bamen Bay > mangrove > river = reservoir. The order of TP (range 0.020–0.876 mg/L) was: aquaculture > Bamen Bay > mangrove > river > reservoir. Meanwhile, the order of TOC (range 4.09–8.68 mg/L) was: aquaculture > mangrove > reservoir > river > Bamen Bay, while the order of COD (range 4.71–13.17 mg/L) was: aquaculture > mangrove > river > Bamen Bay > reservoir. *Chl*.*a*, showed a similar order as COD, and ranged from 19.57 to 64.61 μg/L. δ^2^H and δ^18^O ranged from -47.4‰ to 79.1‰, and -4.3‰ to 12.0‰, respectively. The isotopic order was the same across the water types with: reservoir > Bamen Bay > aquaculture > mangrove > river. Enriched and depleted isotopes were found in the reservoir and river, respectively. The enriched isotopes may have been caused by strong evaporation, while the depletion in the river could have been caused by dilution from multiple tributary water sources.

According to surface water environment quality standards in China [64], pH was in the required range of 6 to 9 for all the water types except for the aquaculture (9.17). DO in the mangrove, with a value of 5.66 (<6.0) belonged to Class II, while DO in the other water types belonged to Class I since they had higher values (>6.0). According to the NH_3_-N values, the river, reservoir, Bamen Bay, mangrove, and aquaculture belonged to Class III, II, IV, IV, and V, respectively. In terms of TP, the river and reservoir belonged to Class III and Class IV, respectively, while the others were inferior in Class V. In terms of the COD_Mn_, the river, reservoir, and Bamen Bay all belonged to Class III, while, the mangrove and aquaculture belonged to Class IV and Class V, respectively. Attention should be paid to the higher NH_3_-N, TP, and COD_Mn_ content in the mangrove and aquaculture waters.

### Spatio-temporal variation of water quality

#### pH, T, EC, DO, and ORP

**Fig 2:**
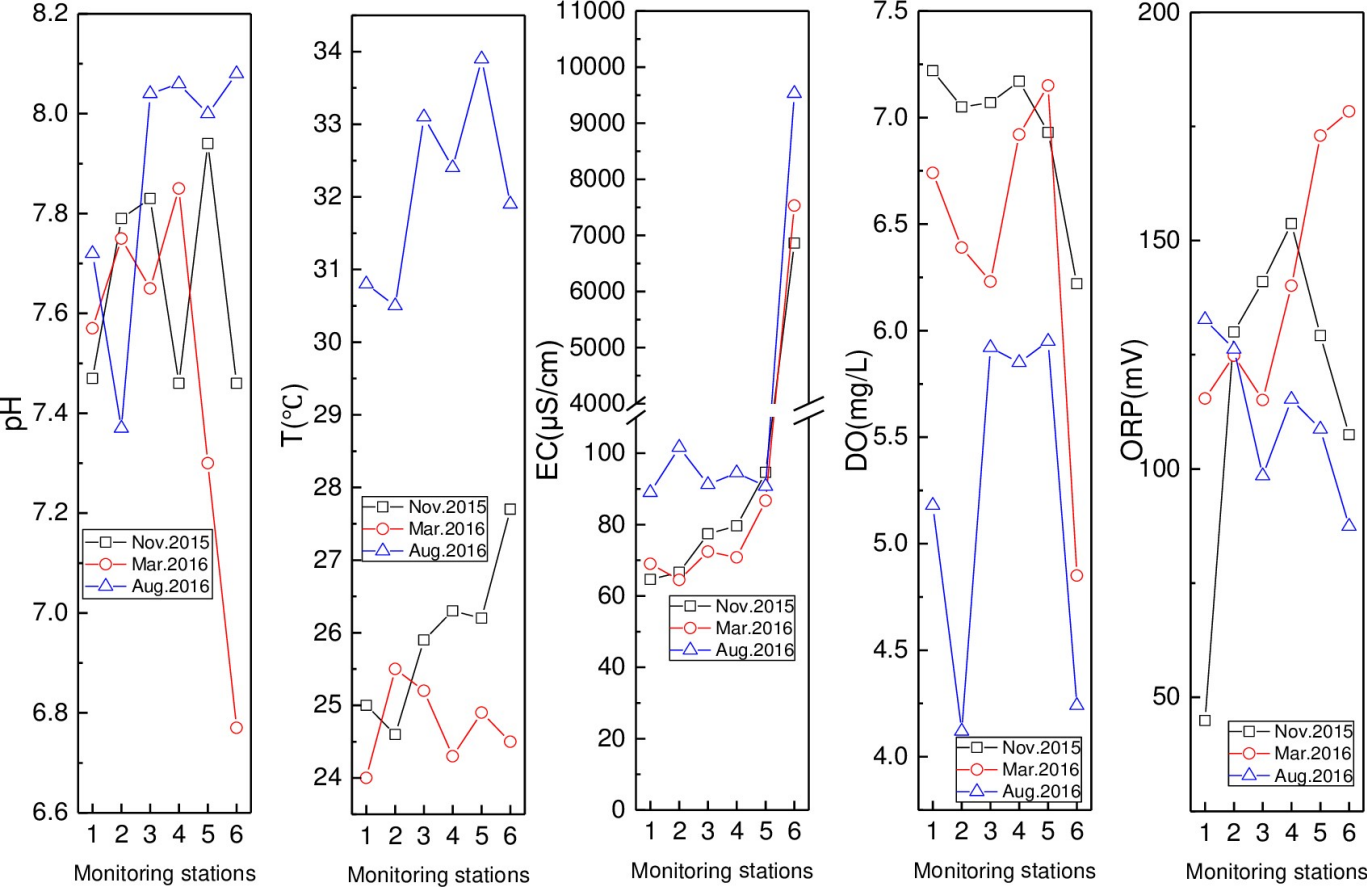
Variation of pH, T, EC, DO, and ORP in Wenchang River (monitoring stations including HN07, HN06, HN05, HN04, HN02, and HN01 were represented by numbers from 1 to 6).

Spatio-temporal variation of pH, T, EC, DO, and ORP in the Wenchang and Wenjiao Rivers is presented in Figs 1 and 2, respectively. A similar spatial pH trend was found in March and November 2015, with the lowest pH value (6.77) found at HN01 in March. The pH initially decreased in August but then increased, with the pH (7.37) at HN02 relatively lower. The pH in all stations was also higher in August than in other locations in other months except at HN07 and HN06. The water temperature in August was the highest, mainly influenced by the air temperature. An initial decrease and then increase in temperature were observed in November, while the opposite trend was seen in March. Spatial variation in EC was consistent over the three months, and a significantly sharp rise from HN02 to HN01, as a result of the impacted of seawater, was observed. EC in August was the highest in the three months. Spatial DO changes were characterized by an initial increase and the decrease trend, that was similar throughout three months. High, medium, and low DO values were measured in November, March, and August, respectively. The lowest and second-lowest values were found in HN06 (4.12 mg/L) and HN01 (4.24 mg/L) in August. The highest and lowest ORP values were found in HN01 (178.3 mV) in March and HN07 (87.4 mV) in November, respectively. Gradual rising and reducing spatial variation in the ORP was observed in March and August, respectively, while, the ORP first increased and then decreased in November.

In Fig 2, the highest (10.57) and second-highest (9.59) pH were found at HN33 (November) and HN11 (March), respectively. The pH and T values in August were higher than those in other months except for the peak pH values. The water temperature (T) in August and March had a similar downward spatial trend, while a slight change was seen in November. The highest and second-highest EC values of 5300 μS/cm and 1303 μS/cm, respectively, were found at HN33 in August and November, in that order. The EC at Barmen Bay was relatively lower upstream than downstream, which probably as a result of seawater intrusions. EC at the first three stations in November were higher than those in March and August. Spatial DO variation was consistent across the three months, showing an initial increase, then decrease, and finally increase again. The highest (9.51 mg/L) and lowest (1.88 mg/L) DO values were observed at HN09 (March) and HN08 (August), respectively. The ORP values in March and August were the highest and lowest, respectively. and showed an initial increase and then decrease spatial trend.

**Fig 3:**
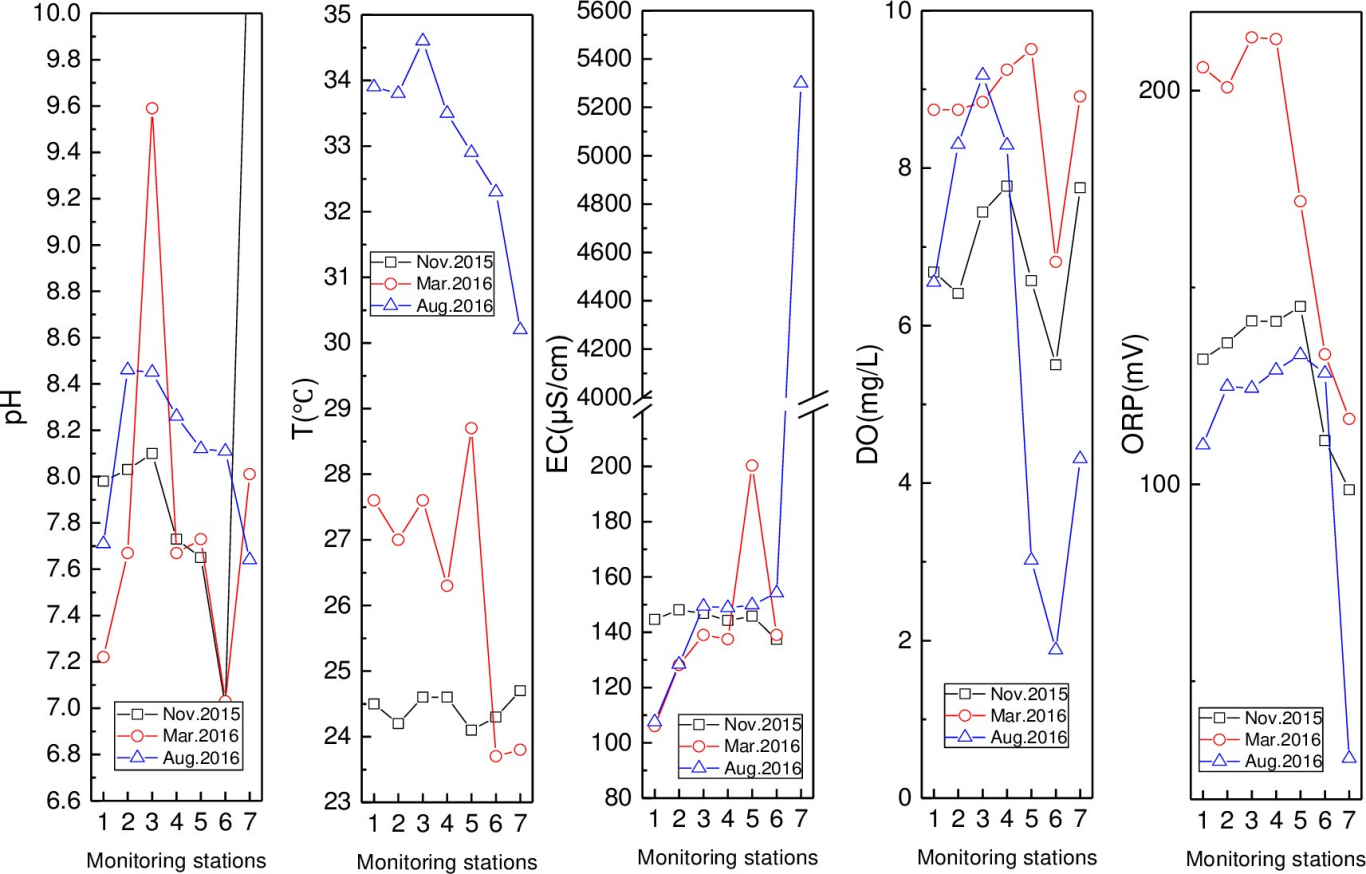
Variation of pH, T, EC, DO, and ORP in Wenjiao River (monitoring stations including HN15, HN14, HN11, HN10, HN09, HN08 and HN33 were represented by numbers from 1 to 7).

The pH, T, EC, DO, and ORP in the reservoir, Bamen Bay, mangrove, and aquaculture are presented in Fig 3. Similar and different temporal pH variations were seen in the reservoir and Bamen Bay, and in the mangrove and aquaculture, respectively. The T and EC were at their highest values in August. The lowest and highest EC values were found in the reservoir and Bamen Bay, respectively. Higher DO was observed in the reservoir, mangrove, and Bamen Bay in August, while a lower DO was measured in the aquaculture. The order of ORP (Bamen Bay > reservoir > aquaculture > mangrove) was similar across the three months. Temporal ORP variations (March > November > August) were consistent in the reservoir, Bamen Bay, and mangrove.

#### Nitrogen, phosphorus, *Chl*.*a*, and COD

**Fig 4:**
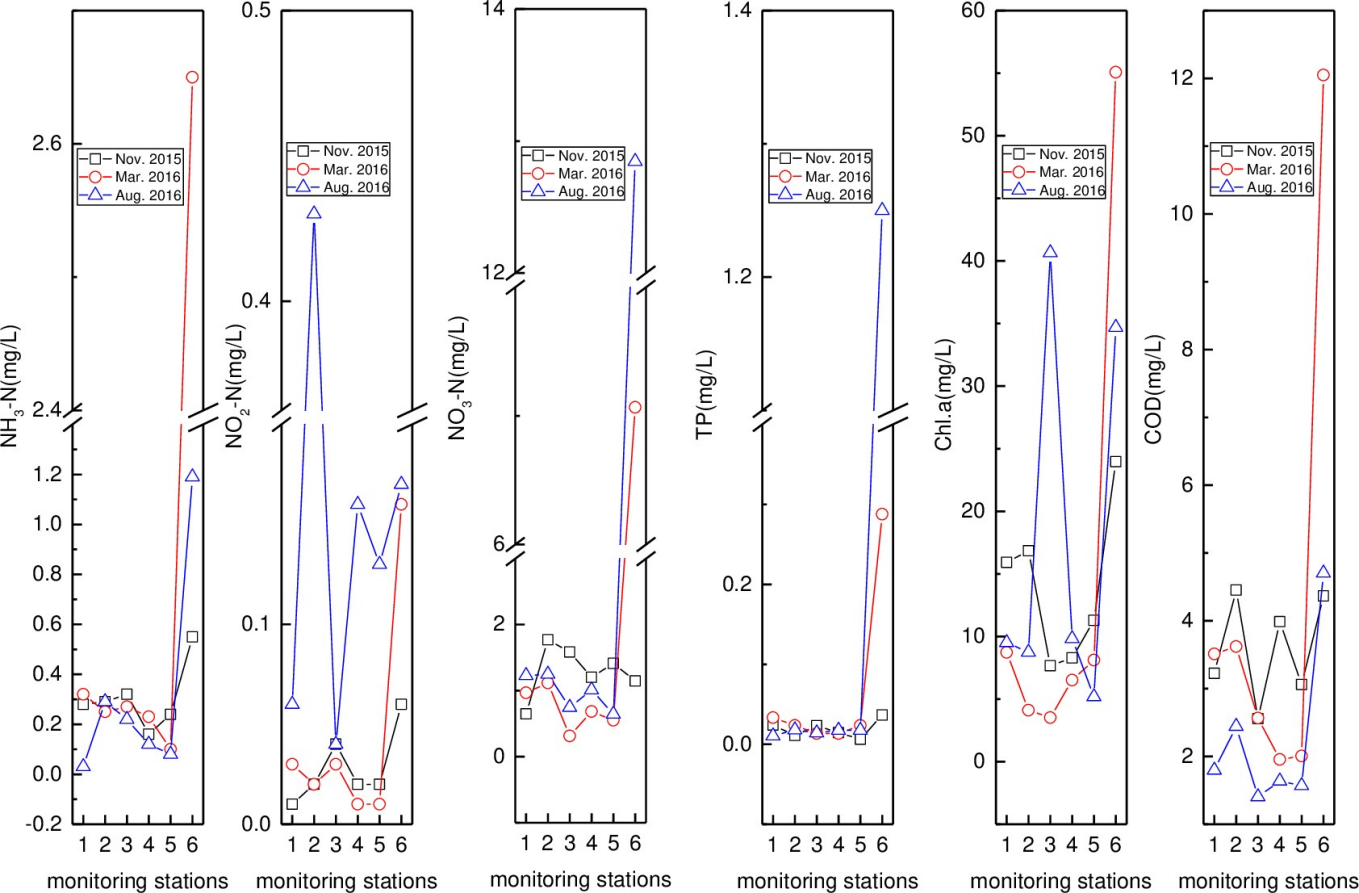
Variation of pH, water Temperature (T), Electrical Conductivity (EC), Dissolved Oxygen (DO) and Oxidation-Reduction Potential (ORP) in the reservoir, Bamen Bay, mangrove, and aquaculture. (Reservoir, Bamen Bay, mangrove, and aquaculture are represented by 1–4).

**Fig 5:**
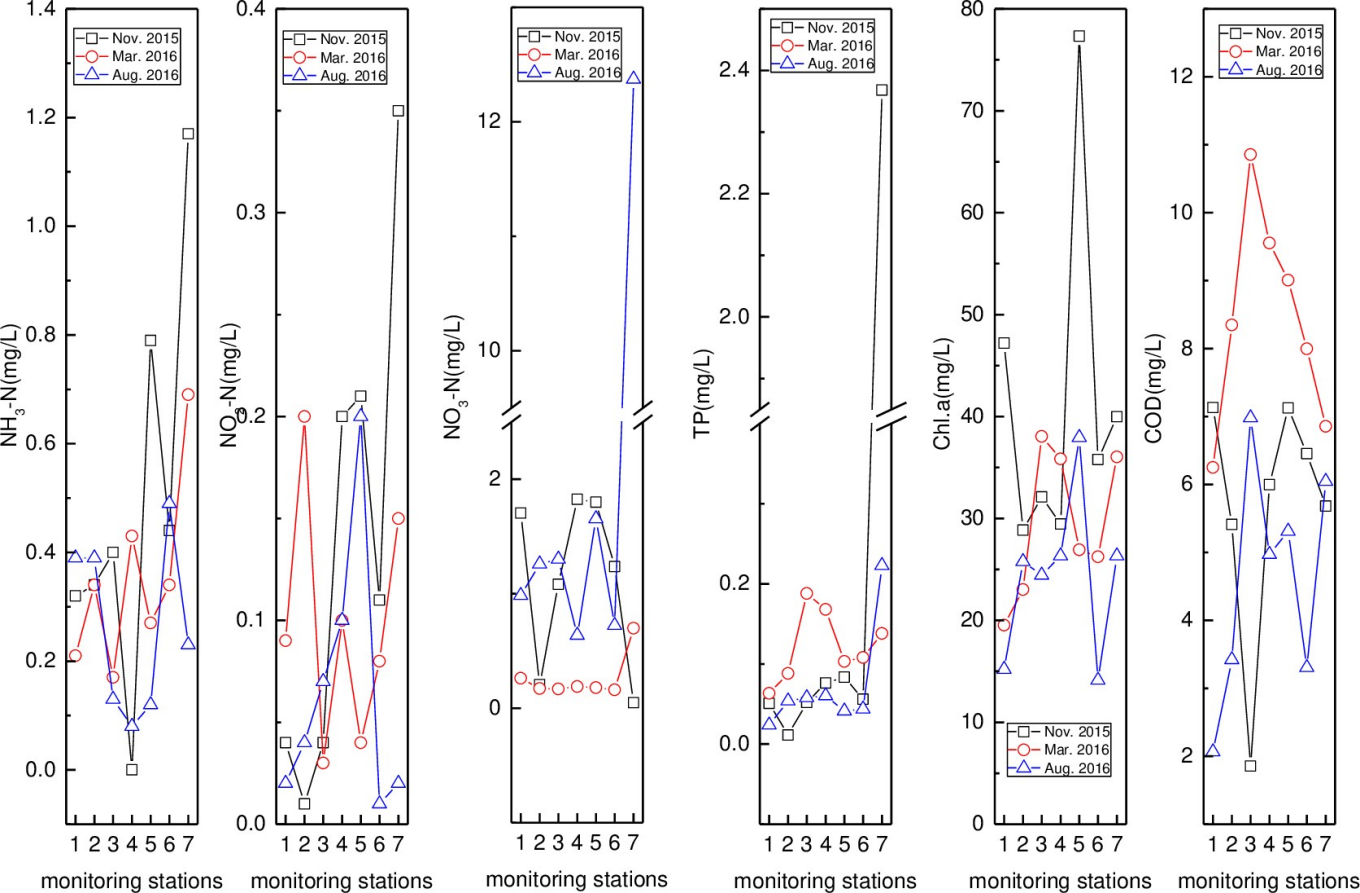
Variation of pH, T, EC, DO, and ORP in Wenchang River (monitoring stations including HN07, HN06, HN05, HN04, HN02, and HN01 are represented by 1–6).

**Fig 6:**
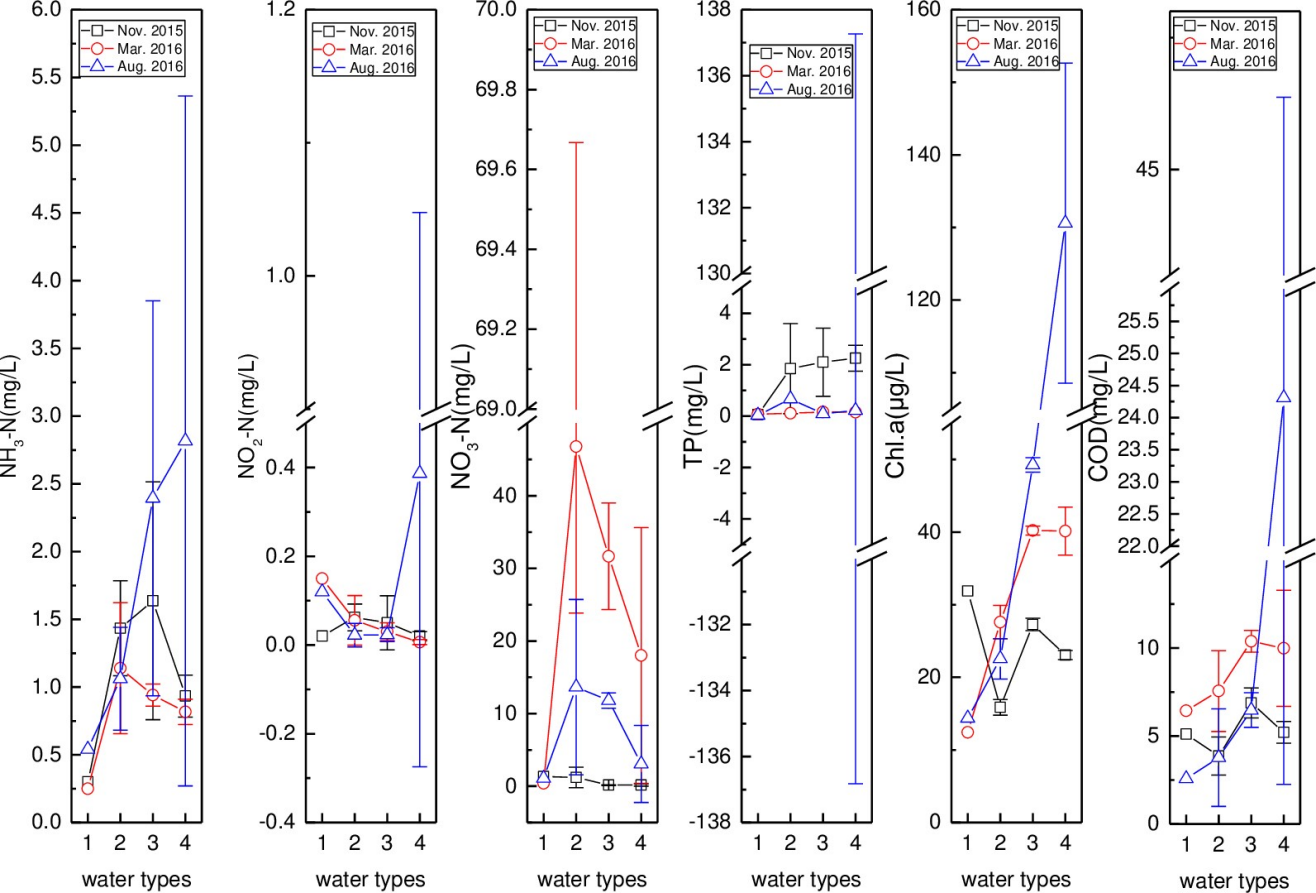
Variation of nitrogen, phosphorus, *Chl*.*a*, and COD in Wenjiao River (names of monitoring stations HN15, HN14, HN11, HN10, HN09, HN08, and HN33 are represented by 1–7).

**Fig 7:**
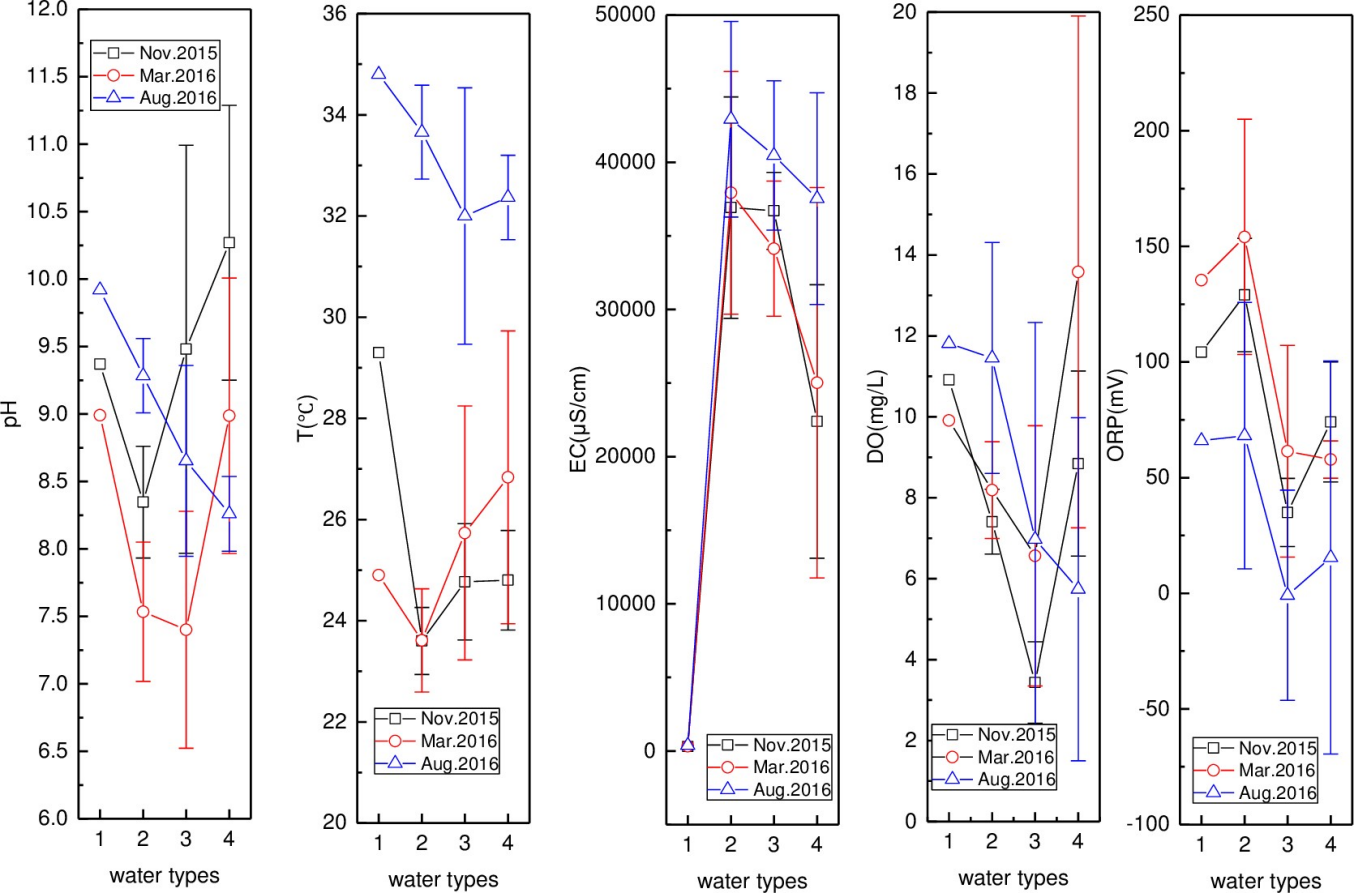
Nitrogen, phosphorus, *Chl*.*a*, and COD in the reservoir, Bamen Bay, mangrove, and aquaculture (these water types are represented by 1–4).

Spatio-temporal variations in nitrogen, phosphorus, *Chl*.*a*, and COD in the Wenchang River, Wenjiao River, and others are shown in Figs 4–6, respectively. In Fig 7, the spatial variation in NH_3_–N was similar across the three months, with the low and high values found at HN07 (August, upstream) and HN01 (March, downstream), respectively. NO_2_–N in August was the highest, with the peak value found at HN06 (August). The NO_3_–N peak (7.07 mg/L) and secondary value (1.15 mg/L) were both found at HN01 (March, August). Meanwhile, NO_3_–N was slightly higher in November than in March and August. The TP content was very low except for the HN01 value, that was 0.037, 0.288, and 1.250 in November, March, and August, respectively. The peak (55.08 μg/L) and secondary value (40.65 μg/L) of *Chl*.*a* were found at HN01 (March) and HN05 (August). The order of COD, except for the peak value, was November > March > August. The peak *Chl*.*a* value on the downstream was consistent with the high nutrient content (NH_3_–N, NO_3_–N, and TP) and COD, resulting in the algae easily reproducing, thereby increasing the chlorophyll content [65]. The peak COD value (12.05 mg/L) was found at HN01 (March), indicating that the organic-matter content at the river and bay intersection was high.

In Fig 4, the peak values of NH_3_–N (1.17 mg/L), NO_2_–N (0.35 mg/L), NO_3_–N (12.37 mg/L), and TP (0.223 mg/L) were all found at HN33 (November) except for NO_3_–N (August). Meanwhile, the lowest values of NH_3_–N, NO_2_–N, NO_3_–N, and TP were found at HN10 (0.00 mg/L, November), HN09 (0.04mg/L, March), HN07 (0.05 mg/L, November), and HN14 (0.011 mg/L, November). The initial decrease and consequential increase were like that of NH_3_–N. The apparent spatio-temporal variability of NO_2_–N was caused by its instability as an intermediate product [66]. Nitrate content was lowest in March, while the values were close in August and November. Meanwhile, the TP was the highest in March, showing that it initially rose and then decreased spatially. Similar trends and a close TP value were observed between August and November. The low TP content was probably caused by the effects of the aquatic plants or algae absorption and dilution. No obvious temporal variation in *Chl*.*a* was observed, with the highest and lowest value observed at HN09 (November) and HN08 (August). COD was higher in March, with the peak (10.86 mg/L) and valley (1.86 mg/L) COD values observed at HN11 (March, November).

The nitrogen, phosphorus, *Chl*.*a*, and COD contents in the reservoir, Bamen Bay, mangrove, and aquaculture are given in Fig 5. Overall, the contents of these parameters over the three months were different across these four water types. Temporal NH_3_–N variations in the reservoir, mangrove, and aquaculture were the same, with August > November > March. However, the NH_3_–N in the Bamen Bay, mangrove, and aquaculture were usually higher than in the reservoir, that had the lowest values (0.25, 0.54, and 0.30 mg/L) in the three months. Meanwhile, the NO_2_–N was very low except for its peak value (aquaculture in August: 0.66 mg/L). The NO_3_–N order over the three months, except for Bamen Bay, was March > August > November. The NO_3_–N contents of these waters were similar in November, with the highest (46.76, 13.65 mg/L) and second-highest (31.67, 11.81 mg/L) values found in the Bamen Bay (March) and mangrove (August), respectively. Low TP values (<1.0 mg/L) were measured in most of the water types except in the aquaculture (2.252 mg/L), mangrove (2.102 mg/L), and Bamen Bay (1.849 mg/L) in November. *Chl*.*a* in the mangrove and aquaculture (March and August) was higher than in the others, with the same temporal variation (August > March > November). The highest (130.61 μg/L) and second-highest (49.26 μg/L) values of *Chl*.*a* were found in the aquaculture and mangrove (August), respectively. Meanwhile, the COD was higher in March except for the peak (24.31 mg/L) measured in the aquaculture in August.

### Ionic relationship and controlling mechanism

#### Gibbs plot

**Fig 8:**
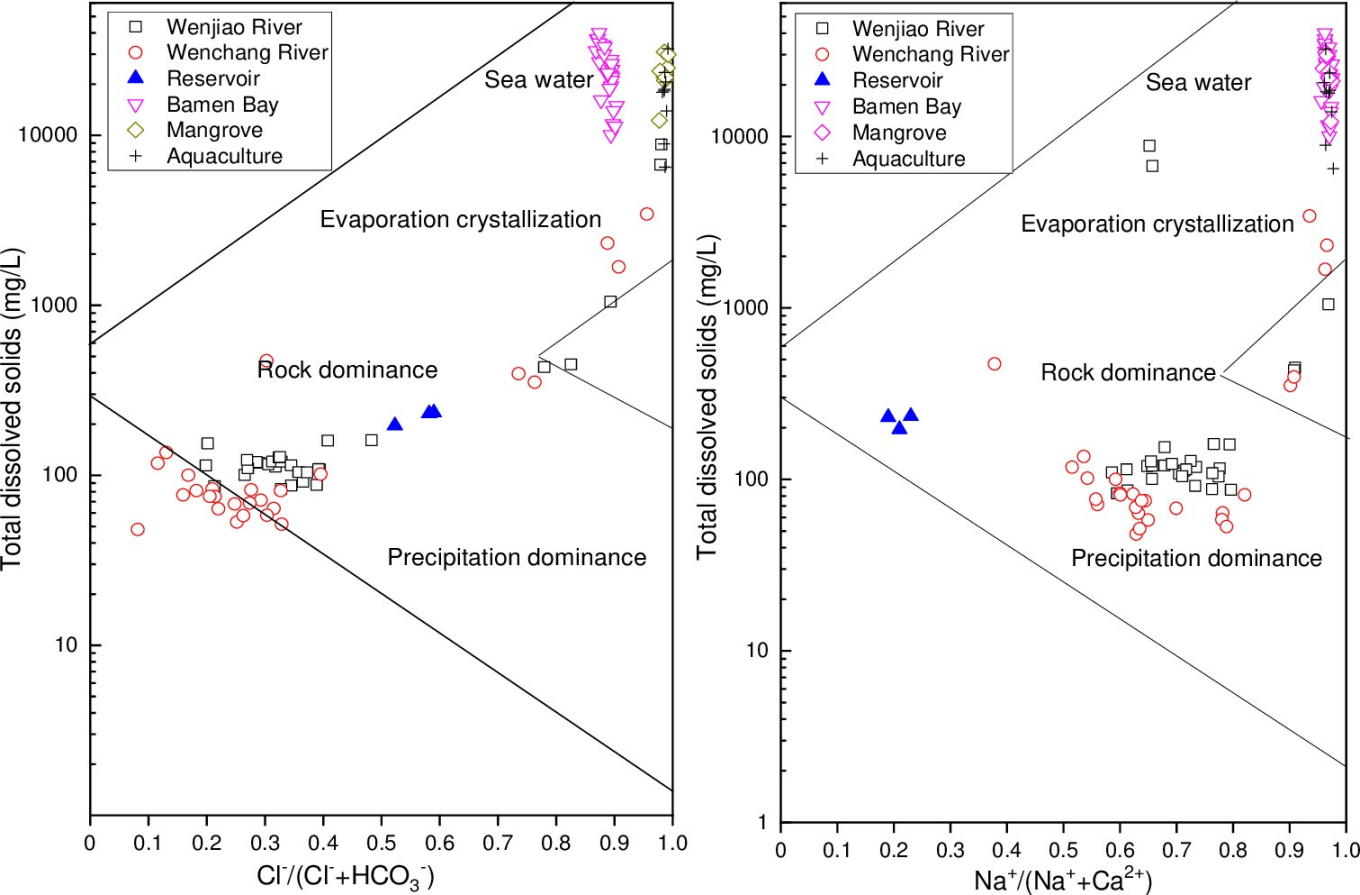
Gibbs plots of the TDS vs. Cl-/(Cl-+HCO3-), and TDS vs. Na+/(Na++Ca2+) of surface water monitoring stations in the Bamen Bay basin.

Gibbs plot can be used to identify the controlling mechanisms in the water chemistry according to the ratio of major ions. The Gibbs plot of surface water monitoring stations in the Bamen Bay basin is given in Fig 8. In summary, the mechanisms governing surface water chemistry consisted of natural factors and anthropogenic activities. Gibbs plots were employed to understand the processes controlling the water chemistry, that included water–rock interactions, atmospheric precipitation, evaporation, and fractional crystallization [4, 67]. The two distal ends of the Gibbs plot were the seawater and precipitation-dominant areas.

Sample distribution was consistent between the cation and anion Gibbs plots. All samples from the Bamen Bay, aquaculture, mangrove, as well as the two samples from the Wenjiao River (HN32 and HN33 in November) fell in the seawater zone of the Gibbs plot. These samples were characterized by a high ion ratio and TDS value. The Cl^-^/(Cl^-^ + HCO_3_^-^) and Na^+^/Na^+^ + Ca^2+^ ratios were higher than 0.90 and 0.96, respectively, while the TDS was around or more than 10,000 mg/L. The two river-monitoring samples showed that the river water was strongly impacted by seawater, especially during the dry season (November), since the monitoring stations were located at the bay mouth. The water chemistry was mainly governed by seawater, although it was a mixture of river fresh water and seawater. Three samples from the Wenchang River (HN16 in March and August, and HN01 in August) and one from the Wenjiao River (HN33 in August) were in the evaporation and crystallization zone. These three sites along the Wenchang River were all located at the mouth of the river into the bay. This indicated the effects of strong evaporation and the influence of mixing with seawater. Meanwhile, the reservoir and most river samples were clustered in the water–rock interaction zone. These samples included those from the Wenchang River (except for five points, HN01 and HN16 in August and November, HN16 in March) and Wenjiao River (except for three samples, HN32 in November, HN33 in August and November). Samples between the interaction zone were relatively close to the precipitation zone, indicating that cations were greatly affected by precipitation, while anions were mainly affected by water–rock interaction. The main land use of the water from the two rivers are cropland and forest [58]. However, samples located in that interaction zone were from monitoring stations away from the bay indicating an enhanced interaction between the soil/rock and surface runoff. River water during the rainy and dry seasons mainly originates from surface runoff and groundwater, respectively. Therefore, river water chemistry was strongly impacted by precipitation and the interaction between runoff or groundwater with the rock or soil. Rock weathering was found to be the dominant controlling factor for the Zhujiang (Guangdong Province, China) water chemistry, with most of the total dissolved load coming from carbonate weathering [68]. The dominant Ca^2+^ and HCO_3_^-^ of the Danjiangkou Reservoir (Hubei Province, China), that belonged to the subtropical monsoon climate region, were also controlled by carbonate weathering [69]. River water chemistry on the upper and lower reaches of the Yellow River were also affected by the weathering of carbonate rocks and the dissolution of evaporites, respectively. On the contrary, rock weathering had almost no effect to the downstream [70]. The effect of rock dominance on the river water chemistry was greater than precipitation in the arid area [7]. Precipitation also played an important role in the subtropical or tropical areas, such as Hainan Province. However, the higher levels of chloride in our research indicate the influence of seawater and anthropogenic activities, since it was not detected in rivers in southern Tibet, which were almost not impacted by human activity [71].

#### Ionic relationship

**Fig 9:**
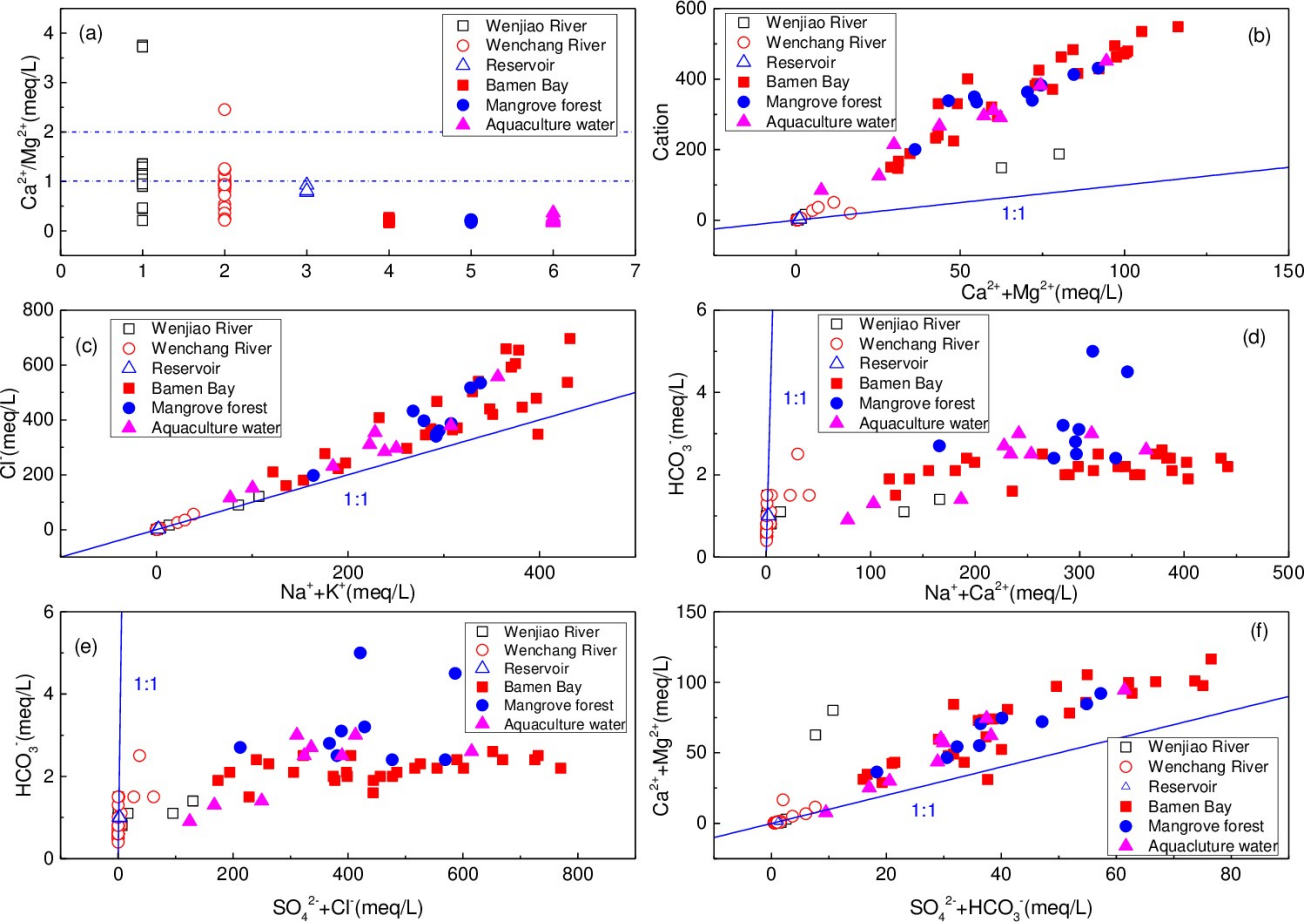
Plots of the ionic relationship of surface water chemistry in the Bamen Bay. (a) Ca^2+^/Mg^2+^, (b) cation vs. Ca^2+^/Mg^2+^, (c) Cl^-^ vs. Na^+^ + K^+^, (d) HCO_3_^-^ vs. Na^+^ + Ca^2+^, (e) HCO_3_^-^ vs. SO_4_^2-^ + Cl^-^, (f) Ca^2+^ + Mg^2+^ vs. SO_4_^2-^ + HCO_3_^-^.

**Fig 10:**
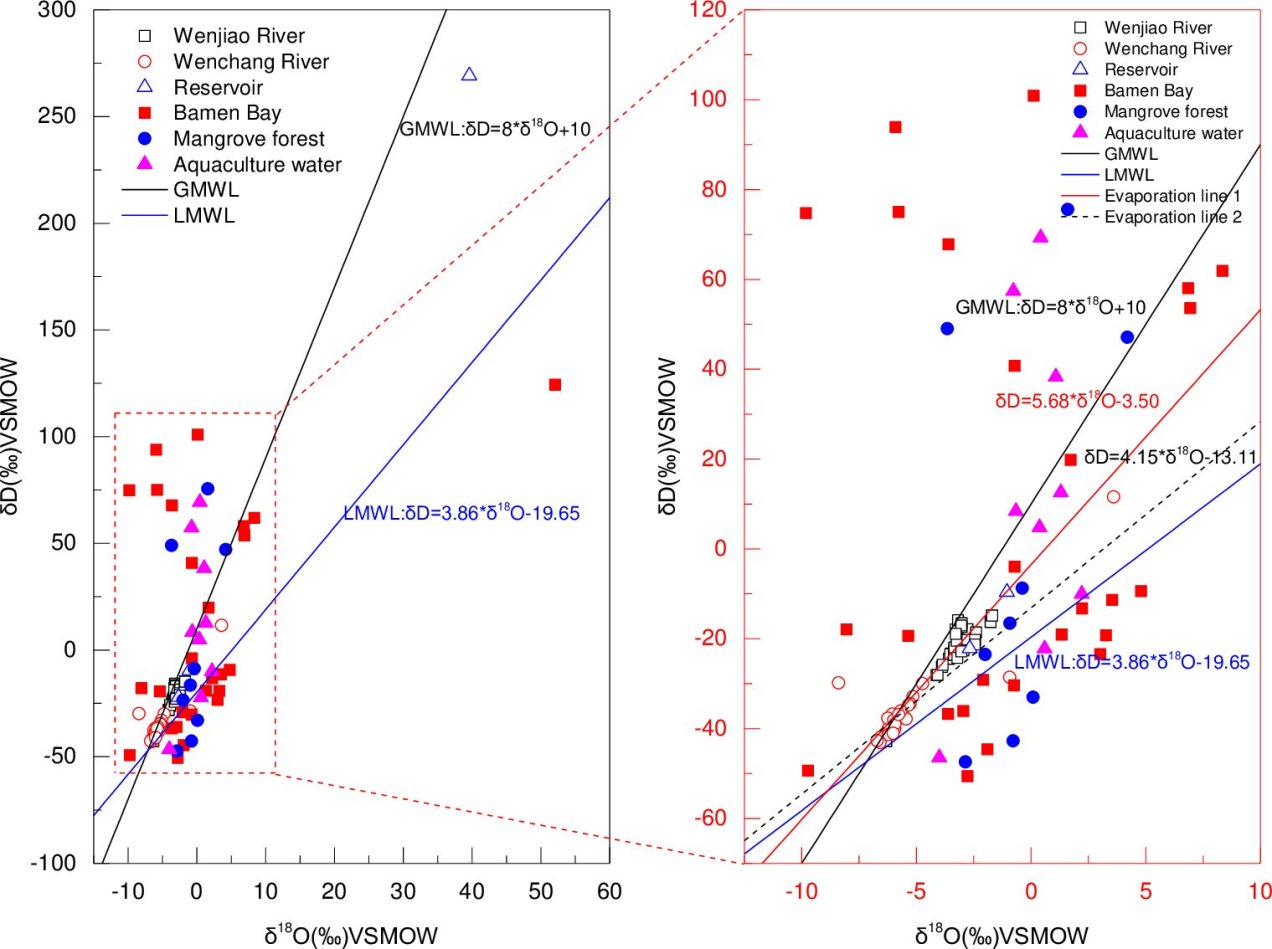
δD–δ^18^O relationships from surface water samples with regression line in Bamen Bay basin. The global and local meteoric water line is also given for reference.

Ionic relationship of surface water chemistry in the Bamen Bay is shown in Fig 8. Most values of Ca^2+^/Mg^2+^ (Fig 9A) were around 1, indicating dolomite dissolution. Meanwhile, the values of three samples (HN32, HN33, and HN05 in November) were higher than 2, indicating excessive Ca^2+^, which may have originated from calcite dissolution and/or anthropogenic sources. Values of Ca^2+^/Mg^2+^ much less than 1 indicated the precipitation of calcium ions [67]. The values of Ca^2+^/Mg^2+^ (Fig 9A) between 1 and 2 obtained for samples HN08, HN09, HN10, HN11, HN14, and HN15 in November; HN08 to HN15 in March and August; HN02, HN06, and HN35 in March; and HN06, HN07, and HN35 in August, indicated calcite dissolution. Meanwhile, three samples (HN32, HN33, and HN05 in November) had values higher than 2, while the remainder had values less than 1. The values of Ca^2+^ + Mg^2+^ vs. cations (Fig 9B) in all water samples were above the balance line (1:1), showing that Ca^2+^ and Mg^2+^ were mainly derived from carbonate weathering and calcite dissolution [72, 73]. In contrast, the value of Na^+^ + K^+^ vs. Cl^-^ (Fig 9C) was all around the balance line, indicating the influence of seawater, strong evaporation, and salt-rock dissolution [74]. The dissolution rate of evaporite was 40–80 times higher than that of granite, and 4–7 times higher than that of carbonate [16]. Meanwhile, most values of HCO_3_^-^ vs. Na^+^ +Ca^2+^ (Fig 9D) were below the balance line, showing that the Na^+^ and Ca^2+^ were higher than HCO_3_^-^. It furtherly indicated the dissolution of calcium-containing minerals. The values of HCO_3_^-^ vs. SO_4_^2-^ + Cl^-^ in most samples (Fig 9E) were below the balance line, indicating the effects of evaporation. This was consistent with the rich δ^18^O isotope (Fig 10). Most samples from the Wenchang River and Wenjiao River were around the line (Fig 9D and 9E), indicating bicarbonate dissolution.

The values of Ca^2+^ + Mg^2+^ vs. HCO_3_^-^ + SO_4_^2-^ (Fig 9F) were located above and near the 1:1 equilibrium line, indicating the presence of excessive Ca^2+^ + Mg^2+^ ions. These excess positive charge must be balanced by other anions like SO_4_^2-^ and/or Cl^-^ (Fig 9C). Meanwhile, cation exchange processes may explain why Na^+^ would replace Ca^2+^/Mg^2+^adsorbed by the rock and soil to form water rich in Ca^2+^/Mg^2+^, when seawater enters freshwater or an aquifer [75]. Therefore, this indicated carbonate and silicate dissolution [76]. The origination and formation mechanism could be inferred from the ionic relationship between major ions in the surface water. Carbonate weathering plays a leading role in river water chemistry in many watersheds around the world, such as, the upper reaches of the Yellow River and Yangtze River [70], since carbonates are more susceptible to weathering than silicates [77].

### Relationship of δD and δ^18^O

Fig 10 shows the relationship of δD and δ^18^O in all surface water samples. The average δ^18^O values of the mangrove, aquaculture, and Bamen Bay were -1.1‰ (-2.9‰ to -0.1‰), 0.1‰ (-4.0‰ to 2.2‰), and -1.0‰ (-9.7‰ to 4.8‰) except for samples in August. The average values of δ^18^O in the Wenjiao and Wenchang River were -3.2‰ (–6.3‰ to -1.7‰) and -5.4‰ (-8.4‰ to -3.6‰), respectively. The slopes of the evaporation line equations of the Wenjiao River ($\delta D\text{=}4.15\text{*}\delta^{18}O\text{-}13.11$) and Wenchang River ($\delta D\text{=}5.68\text{*}\delta^{18}O\text{+}10$) were between the corresponding values of the GMWL (Global meteoric water line) and LMWL (Local meteoric water line). The LMWL was calculated from 12 stations throughout the whole Hainan island from July to August in 1990 [78]. Meanwhile, the δ^18^O of the Bamen Bay water was close to the seawater value (-2 to -3‰) given by the International Atomic Energy Agency (IAEA) [79]. The LMWL slope of 3.86 [78] was lower than that GMWL [80], indicating that water vapor was moved inland from marine source [79]. Furthermore, it showed that river water was mainly derived from atmospheric precipitation. The slopes of the evaporation line equation of the Wenjiao River and Wenchang River were below that of GMWL, which indicated the effect of strong evaporation experienced by river water. However, river water isotopes were enriched comparing to the LMWL. This LMWL was derived from the data 0f 12 monitoring stations from July to August in the Whole Hainan Island [78]. Therefore, it could not fully represent the real LMWL in Bamen Bay catchment. This case could also be inferred from the fact that the slope of evaporation line was higher than that of LMWL in Hainan Island. The isotope difference between the river water and LMWL could be explained by stations throughout Hainan island. Precipitated δ^18^O in low-elevation continental sites in islands, coastal areas, and tropical regions showed similar characteristics to the seawater isotopes, which usually represented the first condensation of undisturbed ocean-water vapor. Therefore, detailed precipitation information of the study area should be furtherly observed, since stable precipitation isotopes in different areas of Hainan Island may have certain differences. Most samples from the mangrove, aquaculture, and Bamen Bay in March and November were around or below GMWL and LMWL, except for the samples in August, which were above the GMWL. This may be due to the mixing of the precipitation, river water, and seawater.

### Nitrogen forms and redox condition

**Fig 11:**
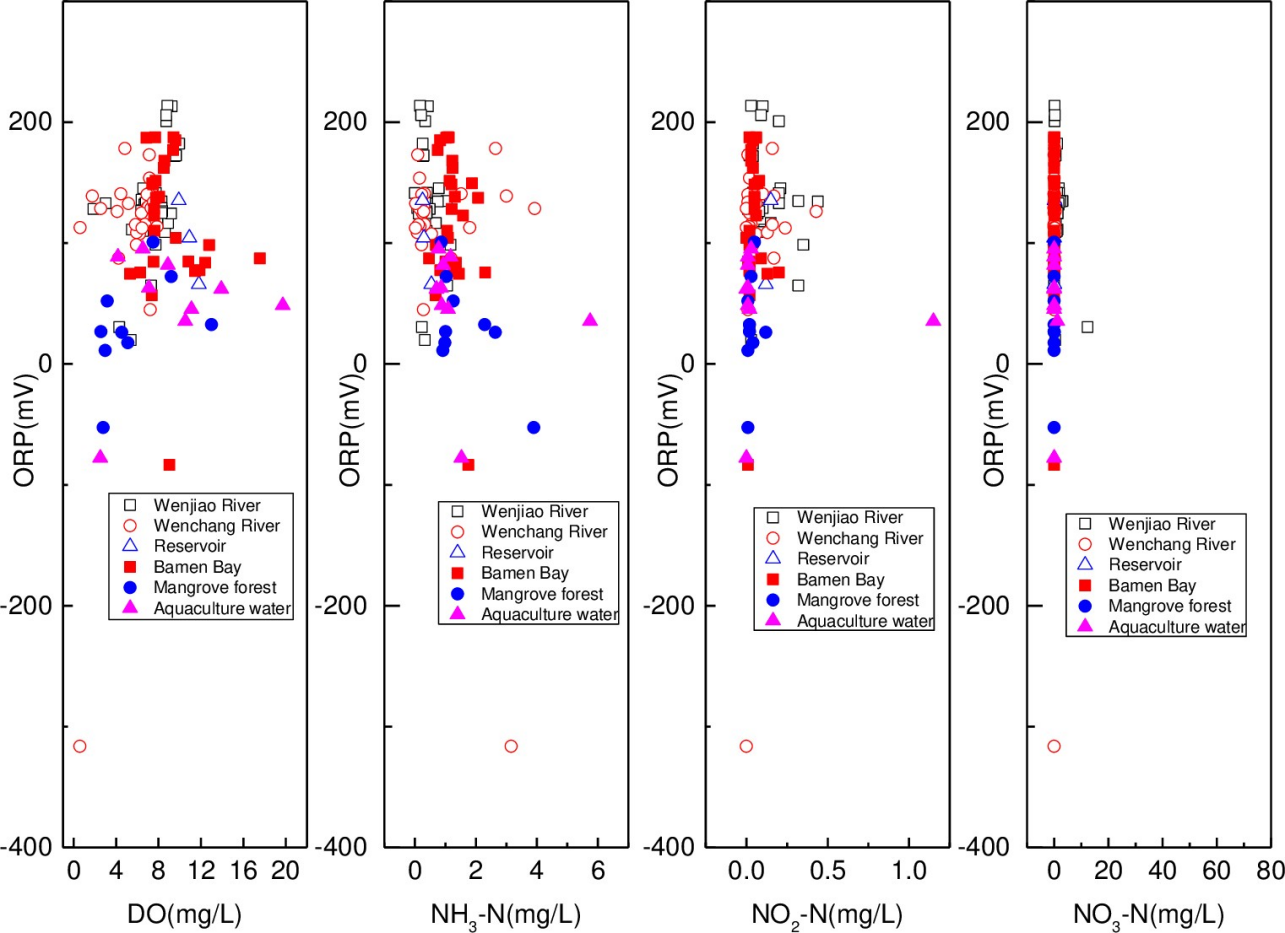
Relationship between ORP and (a) (b) DO, NH_3_–N, (c) NO_2_–N, and (d) NO_3_–N in the Bamen Bay catchment surface water.

In Fig 11, the ORP values were all positive except four negative samples (HN16, HN25, HN26, and HN28 in August). The corresponding NO_2_–N or NO_3_–N content in the positive ORP samples were very low or under detection limit. The average positive ORP value in the Wenjiao River, Wenchang River, reservoir, Bamen Bay, mangrove and aquaculture were 133.7, 124.3, 101.9, 124.8, 42.4, and 64.9 mV, respectively. Correspondingly, the average DO, NH_3_–N, NO_2_–N, and NO_3_–N ranged from 5.76 to 10.88 mg/L, 0.42 to 1.52 mg/L, 0.10 to 0.16 mg/L, and 0.94 to 21.02 mg/L, respectively. ORP in the field was the most important factor that determined the nitrogen forms in the water environment [81, 82]. Corresponding values of positive ORP and nitrogen forms indicated that reducing conditions were not conducive for oxidized nitrogen forms [83, 84]. Mangrove was in the lower reaches of the river and nitrogen, phosphorus, and organic-carbon sink [46, 85], while the aquaculture wastewater had a high organism content [50, 51], and the DO was consumed by the decomposition of organic matter [86]. As a result, lower ORP was found in the mangrove and aquaculture. Redox conditions were positively influenced by the DO content and oxidation ions [87], which could be inferred from consistent DO and ORP values. Denitrification hardly occurred due to the oxidizing condition of the surface water, that was, however, conducive for the mineralization of organic nitrogen and nitrification of ammonium. Nitrite was an intermediate product of nitrification or denitrification [66]. As a result, these reactions could be ascertained by the concentrations of NH_3_–N, NO_2_–N, and NO_3_–N. Furthermore, the different order of reaction intensity could be deduced from variate nitrogen forms, especially nitrate. Except during nitrification, NO_3_–N was also controlled by mixing/dilution and absorption by aquatic plants [88]. Significant negative correlation (S2 Table) of NH_3_–N with DO was found in the Wenchang River and Bamen Bay, while the mangrove showed significant negative correlation with the ORP. This further confirmed that NH_3_–N was easily oxidized via nitrification in an oxidation environment. Meanwhile, significant positive correlation of NH_3_–N with NO_2_–N was observed in the aquaculture and Bamen Bay. This indicated that NO_2_–N was produced as an intermediate product of nitrification [66]. Significant negative and positive correlations of ORP with NO_3_–N and NH_3_–N were found in the Wenjiao and Wenchang River, respectively. The negative correlation indicated that the NO_3_–N was influenced by other factors [88], while the positive correlation was different from the former.

**Fig 12:**
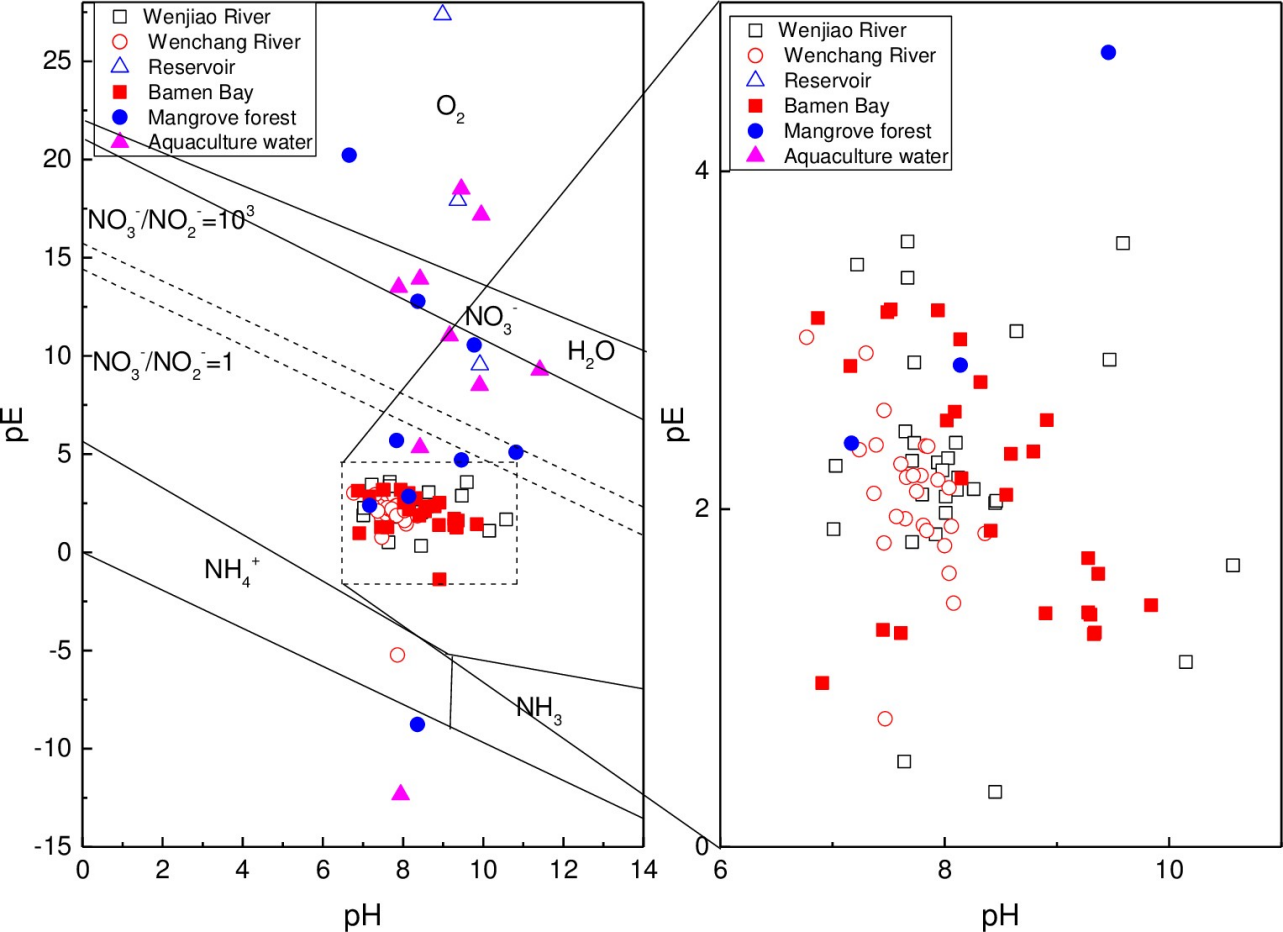
pH–pE diagram of surface water samples in the Bamen Bay watershed.

The combined effects of pH and pE on nitrogen forms are described by the pH–pE diagram, that are shown in Fig 12 for all surface water samples.

Inorganic nitrogen forms in water environment usually include NO_3_^-^(+5), NO_2_^-^(+3), NH_4_^+^(–3), NH_3_ (–3), N_2_O (+1), and N_2_ (0). Most samples in the Wenjiao River, Wenchang River (except one sample), and Bamen Bay were located below the NO_3_^-^/NO_2_^-^ line. This meant that NO_3_^-^ and NO_2_^-^ were the stable nitrogen forms. Three samples were located at the bottom, which included samples from the HN16 in the Wenchang River, HN26 in the mangrove, and HN28 (August) in the aquaculture. Furthermore, eight samples were near the NO_3_^-^/NO_2_^-^ = 10^3^ line, that included samples from HN03 (August) in the reservoir, HN31 (November and March), HN27 (March and August), and HN28 (March) in the aquaculture water, where pH and ORP ranged from 8.42 to 11.41 and 45.2 to 88.7 mV, respectively. Five samples were on the upper area, which included samples from the HN29 (March) in the mangrove, HN03 (March and November) in the reservoir, and HN27 and HN28 (November) in the aquaculture. Oxidized nitrogen was mainly produced by nitrification, which was strongly influenced by pH and DO [89]. DO was consumed by nitrification, while the equilibrium concentration of ammonium and nitrification-enzyme activity were also impacted by pH [84, 90]. As a result, the high ORP oxidation condition was beneficial to nitrification and the formation of nitrite and nitrate. Therefore, nitrate in the samples near the NO_3_^-^/NO_2_^-^ = 10^3^ line was in a potentially stable form [83, 91], hence nitrification was the main controlling factor of oxidized nitrogen forms in these waters. The reduced nitrogen in the samples located at the bottom was potentially the stable form resulting from the reducing conditions, where the ORP value was below zero. Inherently, oxidation hardly occurred under the reducing condition [92], hence no potential stable nitrogen forms were found in the upper area (Fig 11).

## Conclusions

Our research focused on the water chemistry composition and stable isotopes, and determining the surface water controlling factors in the Bamen Bay watershed, where a natural reservation of mangroves is located. The main conclusions are as follows: All the water types were alkaline, and the NO_3_–N was the main inorganic nitrogen form. The order of major inorganic nitrogen forms in different waters was the same: NO_3_–N > NH_3_-N> NO_2_–N. Meanwhile, nitrogen, total phosphorus, TOC, and COD were higher in aquaculture water than in the other waters. However, the stable isotopes δD and δ^18^O were enriched in the reservoir and depleted in river water, showing an influence by evaporation and mixing effects. Most parameters varied dramatically in time and space. Despite this, water chemistry in the river and reservoir were mainly controlled by water–rock interactions and cation exchange, including calcite, bicarbonate, carbonate, and calcium-containing mineral dissolution as deduced from the analysis of the ionic relationship. Mangrove, Bamen Bay, and Aquaculture water were governed by seawater, with the sites close to Bamen Bay also strongly influenced by seawater. Most samples in March and November were around or below the GMWL and LMWL, while the samples in August were located above the GMWL. This may have been caused by the mixing of the precipitation, river water, and seawater. The oxidized condition in the river and Bamen Bay was conducive for nitrification, while the reducing environment in the mangrove and aquaculture water was beneficial for the stability of reduced nitrogen forms.

## Supporting information

S1 Table Statistics of water quality and stable isotope in surface water of the Bamen Bay basin. (DOCX)

S2 Table Pearson correlation among ORP, DO, NH_3_-N, NO_2_-N and NO_3_-N. (DOCX)
