# Supplementary material for: Characteristics of surface water quality and stable isotopes in Bamen Bay watershed, Hainan Province, China
Source: PLoS One. 2021 Jan 22;16(1):e0245438. doi: 10.1371/journal.pone.0245438 (PMC7822266; doi:10.1371/journal.pone.0245438)
Supplement: S1 Table — (DOCX) [file pone.0245438.s001.docx]

S1 Table. Statistics of water quality and stable isotope in surface water of Bamen Bay basin

|  | River | | | | | Reservoir | | | | | Bamen Bay | | | | | | Mangrove | | | | | | aquaculture | | | | | |
| --- | --- | --- | --- | --- | --- | --- | --- | --- | --- | --- | --- | --- | --- | --- | --- | --- | --- | --- | --- | --- | --- | --- | --- | --- | --- | --- | --- | --- |
|  | Mini. | Max. | Mean | SD | C.V. | Mini. | Max. | Mean | SD | C.V. | Mini. | Max. | Mean | SD | C.V. | Mini. | | Max. | Mean | SD | C.V. | Mini. | | Max. | Mean | SD | C.V. |  |
| pH | 6.77 | 10.57 | 7.94 | 0.67 | 8.49 | 8.99 | 9.92 | 9.43 | 0.47 | 4.96 | 6.87 | 9.84 | 8.39 | 0.83 | 9.90 | 6.66 | | 10.82 | 8.51 | 1.31 | 15.37 | 7.89 | | 11.41 | 9.17 | 1.15 | 12.51 |  |
| T(℃) | 23.7 | 34.6 | 27.7 | 3.66 | 13.18 | 24.9 | 34.8 | 29.7 | 4.96 | 16.72 | 21.1 | 34.6 | 27.0 | 4.90 | 18.19 | 23.6 | | 34.9 | 27.5 | 3.88 | 14.12 | 23.5 | | 33.3 | 28.0 | 3.74 | 13.37 |  |
| EC(μS/cm) | 65 | 10980 | 1084 | 2533.78 | 233.66 | 310 | 381 | 337 | 38.21 | 11.33 | 19840 | 48000 | 39257 | 7687.13 | 19.58 | 28900 | | 44200 | 37100 | 4584.76 | 12.36 | 11770 | | 45500 | 28317 | 11296.53 | 39.89 |  |
| ORP(mV) | 316.3 | 213.5 | 121.5 | 69.84 | 57.48 | 66.0 | 135.4 | 101.9 | 34.76 | 34.11 | -83.6 | 187.5 | 117.1 | 57.91 | 49.47 | -52.6 | | 100.8 | 31.9 | 42.75 | 134.19 | -77.7 | | 95.2 | 49.1 | 51.74 | 105.32 |  |
| DO(mg/L) | 0.59 | 9.96 | 6.51 | 2.17 | 33.32 | 9.91 | 11.81 | 10.88 | 0.95 | 8.74 | 5.34 | 17.56 | 9.02 | 2.52 | 27.90 | 2.59 | | 13.01 | 5.66 | 3.58 | 63.28 | 2.51 | | 19.72 | 9.39 | 5.24 | 55.84 |  |
| HCO_3_(mg/L) | 24 | 153 | 55 | 21.89 | 39.95 | 61 | 61 | 61 | 0.00 | 0.00 | 92 | 159 | 132 | 16.43 | 12.47 | 146 | | 305 | 194 | 57.53 | 29.68 | 55 | | 183 | 135 | 48.27 | 35.79 |  |
| Cl(mg/L) | 2.7 | 4302.8 | 243.1 | 754.10 | 310.23 | 66.9 | 87.7 | 79.8 | 11.30 | 14.15 | 5678.6 | 24710.0 | 14773.1 | 5338.81 | 36.14 | 6993.3 | | 18955.2 | 13847.3 | 3574.99 | 25.82 | 4123.6 | | 19754.7 | 10565.8 | 4624.43 | 43.77 |  |
| SO_4_^2-^(mg/L) | 1.1 | 448.1 | 31.7 | 82.48 | 259.96 | 7.8 | 19.9 | 12.2 | 6.72 | 55.13 | 674.6 | 3566.6 | 1935.6 | 867.58 | 44.82 | 752.4 | | 2534.8 | 1731.7 | 580.60 | 33.53 | 411.4 | | 2824.6 | 1347.3 | 692.14 | 51.37 |  |
| K(mg/L) | 1.1 | 159.3 | 11.5 | 26.88 | 233.65 | 12.3 | 17.1 | 14.3 | 2.49 | 17.36 | 99.9 | 393.7 | 244.9 | 82.23 | 33.58 | 132.4 | | 281.3 | 219.0 | 45.94 | 20.98 | 44.5 | | 307.5 | 174.9 | 81.44 | 46.58 |  |
| Na(mg/L) | 5.8 | 2368.0 | 132.0 | 418.73 | 317.15 | 34.2 | 43.0 | 37.3 | 4.98 | 13.35 | 2591.0 | 9719.0 | 6636.9 | 2193.28 | 33.05 | 3693.0 | | 7620.0 | 6426.8 | 1138.09 | 17.71 | 1749.0 | | 8016.0 | 4920.8 | 2002.00 | 40.68 |  |
| Ca(mg/L) | 1.6 | 1263.0 | 47.4 | 209.39 | 441.50 | 7.3 | 10.2 | 9.1 | 1.58 | 17.45 | 90.5 | 382.2 | 226.4 | 86.18 | 38.07 | 103.3 | | 288.8 | 212.2 | 56.31 | 26.53 | 40.7 | | 303.0 | 163.9 | 77.38 | 47.21 |  |
| Mg(mg/L) | 1.1 | 203.8 | 16.6 | 41.25 | 248.32 | 5.6 | 7.3 | 6.5 | 0.84 | 12.92 | 292.3 | 1167.0 | 701.6 | 270.05 | 38.49 | 373.3 | | 931.3 | 653.8 | 187.53 | 28.68 | 67.6 | | 951.7 | 508.1 | 276.42 | 54.40 |  |
| NH_3_-N(mg/L) | 0.00 | 3.93 | 0.60 | 0.81 | 135.85 | 0.25 | 0.54 | 0.36 | 0.16 | 42.67 | 0.47 | 2.31 | 1.21 | 0.43 | 35.10 | 0.87 | | 3.90 | 1.66 | 1.06 | 63.93 | 0.71 | | 5.75 | 1.52 | 1.60 | 105.41 |  |
| NO_2_-N(mg/L) | 0.00 | 0.44 | 0.10 | 0.11 | 112.19 | 0.02 | 0.15 | 0.10 | 0.07 | 70.42 | 0.00 | 0.20 | 0.05 | 0.04 | 90.52 | 0.01 | | 0.12 | 0.03 | 0.04 | 101.73 | 0.00 | | 1.15 | 0.14 | 0.38 | 275.59 |  |
| NO_3_-N(mg/L) | 0.00 | 12.85 | 1.55 | 2.49 | 161.12 | 0.40 | 1.34 | 0.94 | 0.48 | 51.39 | 0.00 | 71.16 | 20.54 | 24.30 | 118.32 | 0.12 | | 37.01 | 14.54 | 14.29 | 98.24 | 0.00 | | 35.84 | 7.08 | 12.39 | 174.98 |  |
| TP(mg/L) | 0.004 | 2.368 | 0.169 | 0.38 | 223.46 | 0.037 | 0.078 | 0.057 | 0.02 | 35.91 | 0.000 | 5.433 | 0.875 | 1.26 | 144.48 | 0.000 | | 3.049 | 0.785 | 1.19 | 151.91 | 0.068 | | 2.717 | 0.876 | 1.07 | 121.88 |  |
| TOC(mg/L) | 0.84 | 12.47 | 5.48 | 3.05 | 55.65 | 7.00 | 7.76 | 7.36 | 0.38 | 5.19 | 1.97 | 25.83 | 4.09 | 4.42 | 108.13 | 5.78 | | 10.93 | 7.88 | 1.70 | 21.60 | 4.60 | | 24.70 | 8.68 | 6.39 | 73.64 |  |
| COD_Mn_(mg/L) | 1.14 | 24.67 | 5.91 | 4.08 | 69.00 | 2.58 | 6.44 | 4.71 | 1.96 | 41.69 | 1.86 | 11.89 | 5.07 | 2.75 | 54.28 | 5.35 | | 10.85 | 7.92 | 2.00 | 25.21 | 1.44 | | 45.46 | 13.17 | 14.09 | 106.99 |  |
| *Chl.a* (μg/L) | 3.51 | 108.01 | 31.15 | 26.19 | 84.10 | 12.43 | 31.89 | 19.57 | 10.72 | 54.78 | 8.38 | 43.15 | 22.00 | 8.78 | 39.90 | 21.19 | | 84.27 | 38.92 | 18.65 | 47.93 | 14.38 | | 281.73 | 64.61 | 85.53 | 132.39 |  |
| δD(‰) | -43.1 | 11.6 | -28.3 | 10.69 | -37.81 | -22.2 | 269.1 | 79.1 | 164.67 | 208.16 | -50.6 | 124.3 | 13.2 | 52.43 | 397.24 | -47.4 | | 75.5 | 0.0 | 45.27 | -126782.41 | -46.5 | | 69.3 | 12.4 | 37.36 | 300.20 |  |
| δ^18^O(‰) | -8.4 | 3.6 | -4.3 | 1.96 | -45.95 | -2.7 | 39.6 | 12.0 | 23.96 | 200.27 | -9.8 | 52.1 | 1.1 | 11.29 | 996.66 | -3.7 | | 4.2 | -0.5 | 2.37 | -453.43 | -4.0 | | 2.2 | 0.1 | 1.78 | 2947.16 |  |
