# Supplementary material for: Characteristics of surface water quality and stable isotopes in Bamen Bay watershed, Hainan Province, China
Source: PLoS One. 2021 Jan 22;16(1):e0245438. doi: 10.1371/journal.pone.0245438 (PMC7822266; doi:10.1371/journal.pone.0245438)
Supplement: S2 Table — (DOCX) [file pone.0245438.s002.docx]

S2 Table. Pearson correlation among ORP, DO, NH_3_-N, NO_2_-N and NO_3_-N

|  | ORP | DO | NH_3_-N | NO_2_-N | NO_3_-N |
| --- | --- | --- | --- | --- | --- |
| Wenjiao River |  |  |  |  |  |
| ORP | 1.000 | 0.440 | -0.076 | 0.433 | **-0.538*** |
| DO |  | 1.000 | 0.032 | 0.155 | -0.359 |
| NH_3_-N |  |  | 1.000 | -0.081 | -1.181 |
| NO_2_-N |  |  |  | 1.000 | -0.257 |
| NO_3_-N |  |  |  |  | 1.000 |
| Wenchang River |  |  |  |  |  |
| ORP | 1.000 | **0.457*** | -0.350 | 0.143 | 0.134 |
| DO |  | 1.000 | **-0.813*** | -0.197 | -0.217 |
| NH_3_-N |  |  | 1.000 | -0.007 | 0.294 |
| NO_2_-N |  |  |  | 1.000 | 0.372 |
| NO_3_-N |  |  |  |  | 1.000 |
| Reservoir |  |  |  |  |  |
| ORP | 1.000 | -0.996 | -0.955 | 0.162 | -0.657 |
| DO |  | 1.000 | 0.924 | -0.250 | 0.722 |
| NH_3_-N |  |  | 1.000 | 0.139 | 0.403 |
| NO_2_-N |  |  |  |  | -0.850 |
| NO_3_-N |  |  |  |  | 1.000 |
| Bamen Bay |  |  |  |  |  |
| ORP | 1.000 | -0.186 | -0.189 | 0.065 | 0.302 |
| DO |  | 1.000 | **-0.514**** | -0.313 | 0.054 |
| NH_3_-N |  |  | 1.000 | **0.466*** | -0.368 |
| NO_2_-N |  |  |  |  | -0.203 |
| NO_3_-N |  |  |  |  | 1.000 |
| Mangrove |  |  |  |  |  |
| ORP | 1.000 | 0.452 | **-0.701*** | 0.206 | 0.350 |
| DO |  | 1.000 | -0.041 | 0.050 | 0.270 |
| NH_3_-N |  |  | 1.000 | 0.148 | -0.380 |
| NO_2_-N |  |  |  | 1.000 | -0.182 |
| NO_3_-N |  |  |  |  | 1.000 |
| Aquaculture water |  |  |  |  |  |
| ORP | 1.000 | 0.238 | -0.218 | -0.087 | 0.171 |
| DO |  | 1.000 | -0.007 | 0.081 | 0.102 |
| NH_3_-N |  |  | 1.000 | **0.987*** | -0.246 |
| NO_2_-N |  |  |  | 1.000 | -0.216 |
| NO_3_-N |  |  |  |  | 1.000 |

*. Correlation is significant at the 0.05 level (2-tailed).

*.*. Correlation is significant at the 0.01 level (2-tailed).
